# MSP-HTPrimer: a high-throughput primer design tool to improve assay design for DNA methylation analysis in epigenetics

**DOI:** 10.1186/s13148-016-0269-3

**Published:** 2016-09-21

**Authors:** Ram Vinay Pandey, Walter Pulverer, Rainer Kallmeyer, Gabriel Beikircher, Stephan Pabinger, Albert Kriegner, Andreas Weinhäusel

**Affiliations:** 1 Health and Environment Department, Molecular Diagnostics, Austrian Institute of Technology GmbH, Vienna, Austria; 2 Institut für Populationsgenetik, Vetmeduni Vienna, Veterinärplatz 1, Vienna, A-1210 Austria

**Keywords:** PCR, Primer design, DNA methylation, Epigenetics, High throughput, CpG islands, Bisulfite deamination, MSRE-PCR, MSP, BSP, COBRA, Pyrosequencing, Targeted bisulfite sequencing

## Abstract

**Background:**

Bisulfite (BS) conversion-based and methylation-sensitive restriction enzyme (MSRE)-based PCR methods have been the most commonly used techniques for locus-specific DNA methylation analysis. However, both methods have advantages and limitations. Thus, an integrated approach would be extremely useful to quantify the DNA methylation status successfully with great sensitivity and specificity. Designing specific and optimized primers for target regions is the most critical and challenging step in obtaining the adequate DNA methylation results using PCR-based methods. Currently, no integrated, optimized, and high-throughput methylation-specific primer design software methods are available for both BS- and MSRE-based methods. Therefore an integrated, powerful, and easy-to-use methylation-specific primer design pipeline with great accuracy and success rate will be very useful.

**Results:**

We have developed a new web-based pipeline, called MSP-HTPrimer, to design primers pairs for MSP, BSP, pyrosequencing, COBRA, and MSRE assays on both genomic strands. First, our pipeline converts all target sequences into bisulfite-treated templates for both forward and reverse strand and designs all possible primer pairs, followed by filtering for single nucleotide polymorphisms (SNPs) and known repeat regions. Next, each primer pairs are annotated with the upstream and downstream RefSeq genes, CpG island, and cut sites (for COBRA and MSRE). Finally, MSP-HTPrimer selects specific primers from both strands based on custom and user-defined hierarchical selection criteria. MSP-HTPrimer produces a primer pair summary output table in TXT and HTML format for display and UCSC custom tracks for resulting primer pairs in GTF format.

**Conclusions:**

MSP-HTPrimer is an integrated, web-based, and high-throughput pipeline and has no limitation on the number and size of target sequences and designs MSP, BSP, pyrosequencing, COBRA, and MSRE assays. It is the only pipeline, which automatically designs primers on both genomic strands to increase the success rate. It is a standalone web-based pipeline, which is fully configured within a virtual machine and thus can be readily used without any configuration. We have experimentally validated primer pairs designed by our pipeline and shown a very high success rate of primer pairs: out of 66 BSP primer pairs, 63 were successfully validated without any further optimization step and using the same qPCR conditions. The MSP-HTPrimer pipeline is freely available from http://sourceforge.net/p/msp-htprimer.

**Electronic supplementary material:**

The online version of this article (doi:10.1186/s13148-016-0269-3) contains supplementary material, which is available to authorized users.

## Background

DNA methylation is an epigenetic mechanism of gene regulation in mammalian genomes, and aberrant methylation has been associated with various biological processes including X-chromosome inactivation [1–4], gene imprinting [5–10], embryogenesis [11, 12], and cancer [13–16]. DNA methylation is a chemically stable key player in epigenetic and heritable over many generations of cell divisions [17]. It is one of the well-known endogenous modifications of DNA in mammals and refers to the enzymatic, post-synthetic addition of a methyl group to the carbon 5 position of the cytosine ring [18]. Bisulfite-based methods and methylation-sensitive restriction enzyme-based PCR (MSRE-PCR) methods have been widely used for detection of DNA methylation. The bisulfite conversion-based PCR methods, such as bisulfite sequencing PCR (BSP), methylation-specific PCR (MSP), COBRA, and MSRE-PCR, are commonly used techniques for methylation detection [19]. In bisulfite conversion-based PCR methods, genomic DNA is treated with bisulfite to convert non-methylated cytosine to uracil by deamination while leaving methylated cytosine unaffected. After deamination with bisulfite, DNA complementary is lost, resulting in single-stranded DNA. Four different sequences can now be found, representing the methylated as well as the unmethylated allele for both the plus and the minus strand. However, this procedure has several limitations, like low multiplexing capability, the inability to discriminate between 5-methylcytosine and 5-hydroxymethylcyosine, the degradation of DNA during bisulfite treatment, high experimental time, and the possibility of incomplete conversion under not ideal reaction parameters. In addition, the harsh conditions in combination with the extreme DNA sequence composition after bisulfite modification make primer design for this type of PCR challenging. Methylation-sensitive restriction enzyme-based PCR (MSRE-PCR) can be used for the rapid, simultaneous detection of DNA methylation in multiple fragments when only a limited amount of DNA is available. It is a procedure based on the fact that digestion of genomic DNA with methylation-sensitive restriction enzymes is blocked when methylated. Best suited for that analyses targeting 5-methylcytosine are enzymes, which contain CpG motifs in their recognition [20, 21]. The MSRE-PCR-based method allows for a high level of multiplexing with manageable efforts regarding assay optimization, and only a few nanograms of DNA (10–20 ng) is needed per 100 assays [22].

Both BS-based as well as MSRE-based methods have some advantage and limitations; however, depending on the requirements, either one or both can be used efficiently by complementing each other. Thus, an integrated pipeline, which can design assays based on both methods under single platform, would be greatly useful for DNA methylation analysis of several genes in a time-effective manner.

Several software tools such as Methyl Primer Express (http://www.appliedbiosystems.com/), MethPrimer [23], BiSearch [24], MethMaker [25], MSPprimer [26], and Bisprimer [27] are available for this purpose. These tools allow users to customize primer length, amplicon length, and Tm (melting temperature) differences, as well as enable searches for CpG islands in the input sequence. But all have several limitations such as the following: (1) do not design on reverse strand, (2) not suitable for high-throughput genome-wide primer design, (3) do not take single nucleotide polymorphism (SNP) [28] in consideration as primers should not bind to regions containing common SNPs [29, 30]; (4) not taking repeat regions in consideration which can cause the formation of hairpins that interfere with proper annealing to the template [31, 32], (5) do not support requirement-based automatic primer design optimization and selection feature, and (6) do not provide genomic and epigenetic annotation for each resulting primers. Therefore, a primer design tool for overcoming these limitations and to design specific, optimized primers with a great success rate in a high-throughput manner for genome-wide DNA methylation analysis would be very helpful for researchers in that field.

We have therefore developed MSP-HTPrimer, an open source, web-based high-throughput, and genome-wide primer design pipeline for MSRE-PCR assay and bisulfite-based assays (MSP, BSP, and COBRA), which is capable of simultaneously processing hundreds to thousands of target sequences. To achieve that goal, we have adapted the current Primer3 primer design process and added genomic annotations, multiprocessing computational capabilities, and new primer selection possibilities. Unlike other tools, MSP-HTPrimer takes genome-wide annotations of SNPs and repeats into consideration to design primer pairs to achieve high success rate. MSP-HTPrimer enables hierarchical filtering and visualization of designed primers in UCSC genome browser for efficient selection of assays [33]. In order to provide a one-stop solution for both BS and MSRE methods under MSP-HTPrimer pipeline, we have integrated the MSRE-PCR tool, which is available as a separate tool and described elsewhere (Pandey et al., Clin Epigenetics. 2016). Thus, MSP-HTPrimer facilitates the design of primers for BSP, MSP, COBRA, and MSRE assays.

## Results and discussion

**Fig. 1:**
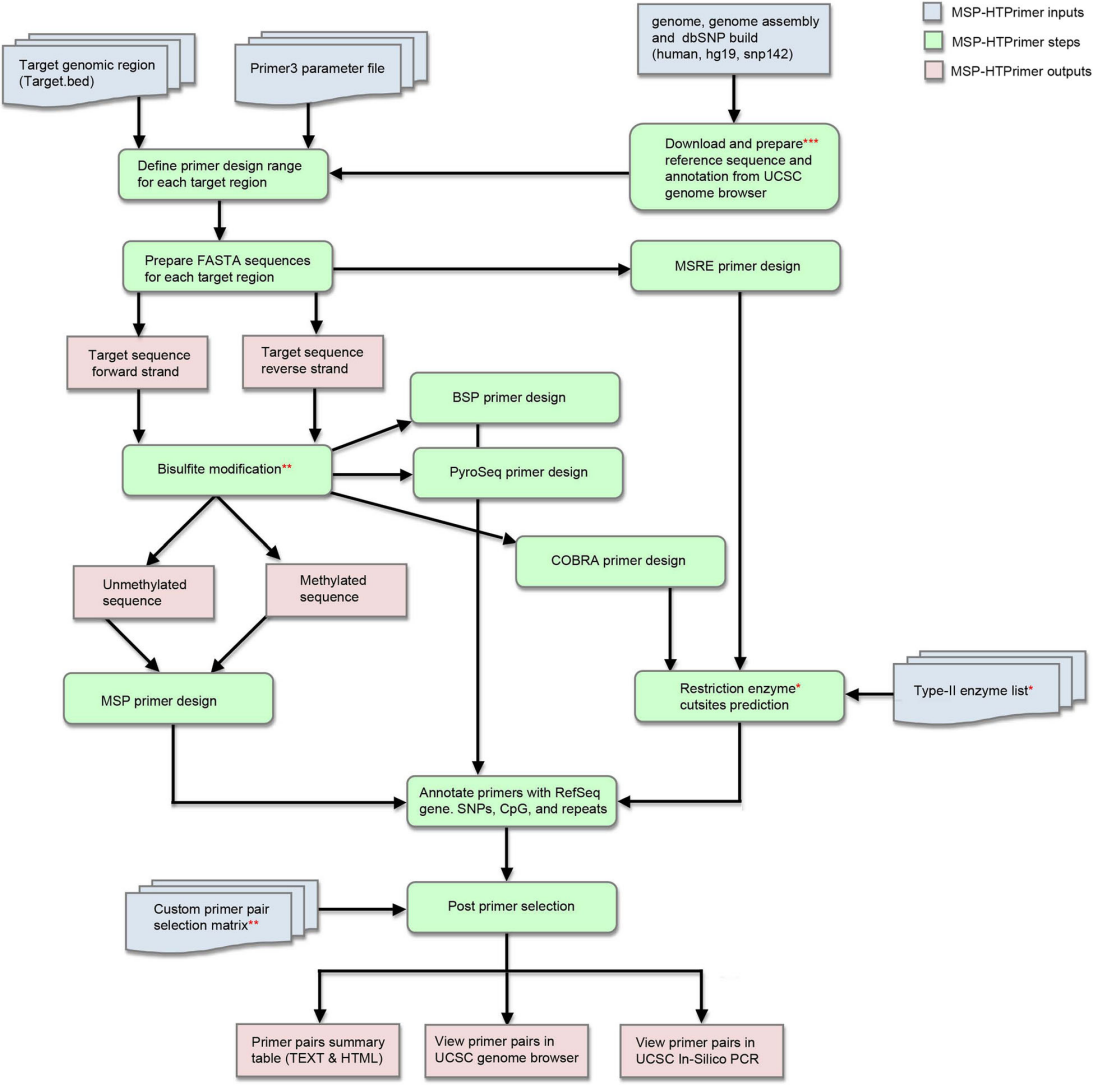
The workflow of MSP-HTPrimer pipeline. The MSP-HTPrimer pipeline can be run via an intuitive web interface. The sequential analysis steps are displayed from top to bottom. The pipeline inputs are depicted in *blue color*, steps are in *green*, and the end outputs are in *red color*. *Restriction enzyme cut site prediction step and the *type-II restriction enzymes list input are only applicable for MSRE-PCR and COBRA-PCR primers. **Bisulfite modification is only applicable for BSP, MSP, and COBRA methods. **Quality filter table file is optional; if user-defined primer selection criteria are not provided, then all primer pairs will be recorded in the final output summary table. ***Download and preparation of the reference sequence and annotation from UCSC genome browser step runs only once

MSP-HTPrimer is an open source, portable, web-based, and easy-to-use pipeline, which facilitates the design of primer pairs for DNA methylation assay design. It uses a simple input format and produces a single output summary table and can design primers for hundreds to thousands of target sequences in a single run. MSP-HTPrimer provides significant improvements over existing solutions with following unique features: (1) automatically designs primers for BSP, BSP-COBRA, and MSP assays on forward and reverse strand, (2) parallel primer design for several target sequences, (3) consideration of SNP and repeats during primer design and selection, (4) hierarchical primer selection and filtering based on custom quality matrix, and (5) visualization of primer pairs in the UCSC genome browser [33]. The pipeline is equipped with multiprocessing computational capability and uses custom inputs and parameters to design specific primers. All components of the MSP-HTPrimer pipeline workflow, inputs, and outputs have been summarized in Fig. 1.

### MSP-HTPrimer pipeline

The MSP-HTPrimer pipeline (Fig. 1) consists of eight sequential steps. Based on a user-defined list of target regions and design parameters, the pipeline retrieves a list of annotated primer pairs (in TXT and HTML format) and creates links to visualize results in the UCSC genome browser. All steps of the pipeline are described as follows:

1. Download and prepare reference sequence and annotation from UCSC genome browser
  During the first primer design process, MSP-HTPrimer downloads and prepares the reference FASTA sequence, common SNPs, RefSeq gene, CpG islands, and annotations of known repeat elements for the entire genome (human and mouse) based on the selected genome, genome assembly, and dbSNP build number from the UCSC genome browser. The default genome is human, genome assembly is hg19, and the dbSNP build number is 142. MSP-HTPrimer does not re-download reference data for subsequent analysis runs. The query interface allows the user to customize all primer design and selection parameters for BSP-PCR, pyrosequencing primer design, COBRA-PCR, MSP-PCR, and MSRE-PCR.
2. Define primer design range for each target region
  In this step, the genomic primer design range is prepared by adding the number of flanking upstream and downstream base pairs (optional) to the actual target region given as input in the target bed file (Additional file 1).
3. Prepare FASTA sequence for each target region
  Target sequences are extracted from the genome reference FASTA file based on the target chromosomal positions. For BS-based methods (BSP, MSP, COBRA), the in silico deduced sequences for methylated and unmethylated alleles from the plus and the minus strand are used for assay design to increase the success rate. Next, MSP-HTPrimer subsets the annotation files based on target region coordinates in order to improve execution time.
4. Bisulfite modification
  In silico “bisulfite”-treated DNA sequences for the methylated and the unmethylated alleles on both strands are generated and subjected to the design software. The software uses for BSP and COBRA design the methylated sequences and for MSP the methylated and the unmethylated sequences (Fig. 2). The MSP design returns one assay for the methylated allele and one assay for the unmethylated allele.

**Fig. 2:**
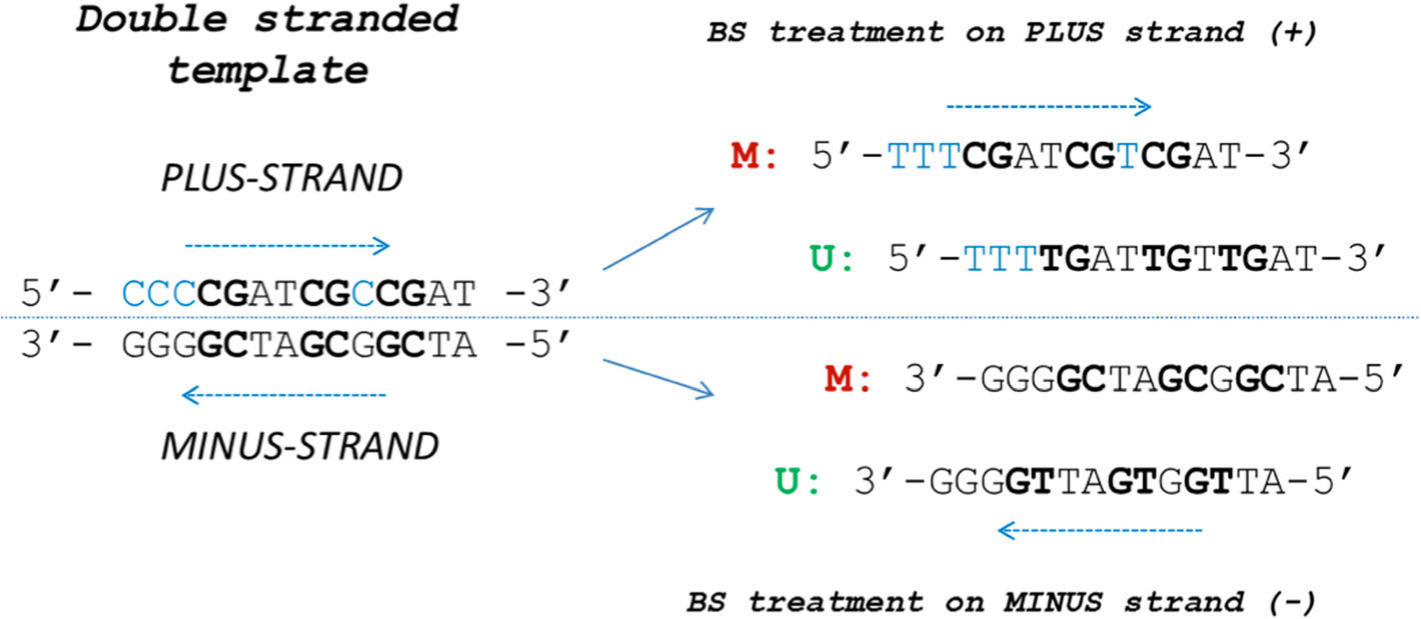
The MSP-HTPrimer sequence: the principle of in silico deamination workflow. The MSP-HTPrimer pipeline takes into account that deamination of double-stranded native DNA (*left*) results in four different template sequences (*right*) for primer design. Thus, from the plus and minus strand, different methylation-dependent primer pairs can be designed (M - methylated; U - unmethylated; Cs in blue - not in a CpG context are usually unmethylated)
5. Primer design with Primer3
  In this step, the tool takes two inputs: (1) FASTA sequences (forward and reverse strand for BSP, MSP, and COBRA assays) from the previous step and (2) a Primer3 parameter input file (Additional file 2). It runs the Primer3 tool to design all possible PCR primer pairs and hybridization probes for all target sequences and stores all resulting primers, probes, and amplicons. MSRE primers are designed with the unconverted, native DNA target sequences (without bisulfite modification), whereas for BSP, PyroSeq, and COBRA methylated target sequences are used as template to Primer3. Since MSP-HTPrimer considers both strands for primer design, thus user can also design the pyrosequencing BSP primer design by adjusting the primer and product lengths and melting temperature [34]. For MSP, primer pairs are designed to specifically amplify either methylated or unmethylated DNA [35]. In MSP assay, the methylated primer set assumes that the CpGs are fully methylated. Therefore, the primer will have all four bases in the sequence. On the other hand, the unmethylated primer set anneals to unmethylated DNA in the (same) primer binding site, and therefore it will have T in place of C in the primer sequence [35].
6. Restriction enzyme cut site prediction
  In this step, the enzyme’s cut sites in amplicons are predicted. This step is only applicable for MSRE-PCR primer design and COBRA-PCR. COBRA primer design is similar to BSP, where amplicons should contain at least one cut site but no CpGs, and should contain several “Cs” which will amplify the bisulfite deaminated sequence and not the native DNA.
7. Annotate each primer pair with gene, SNP, CpG, RefSeq genes, and repeats
  In this step, each primer pair, hybridization probe, and amplicon is annotated with RefSeq gene-IDs found in a 1-kb region upstream and downstream of the target region, SNPs, CpG islands, and repeat regions. These annotations will help to pick the suitable primer pairs for each target region.
8. Primer selection
  Based on the user-defined selection criteria, the final primer pairs for each target region are selected. These selection criteria input file is optional (see Additional file 3). This step facilitates selection of primer candidates according to the filtering criteria and provides the specific primer pairs for hundreds of target regions in a time-effective manner. This customized hierarchical filtering process is a unique and very useful feature of the MSP-HTPrimer tool, which is lacking in all other freely available tools. Finally, MSP-HTPrimer produces a primer summary table in TXT and HTML format and allows visualization of results in the UCSC genome browser.

### Query interface

**Fig. 3:**
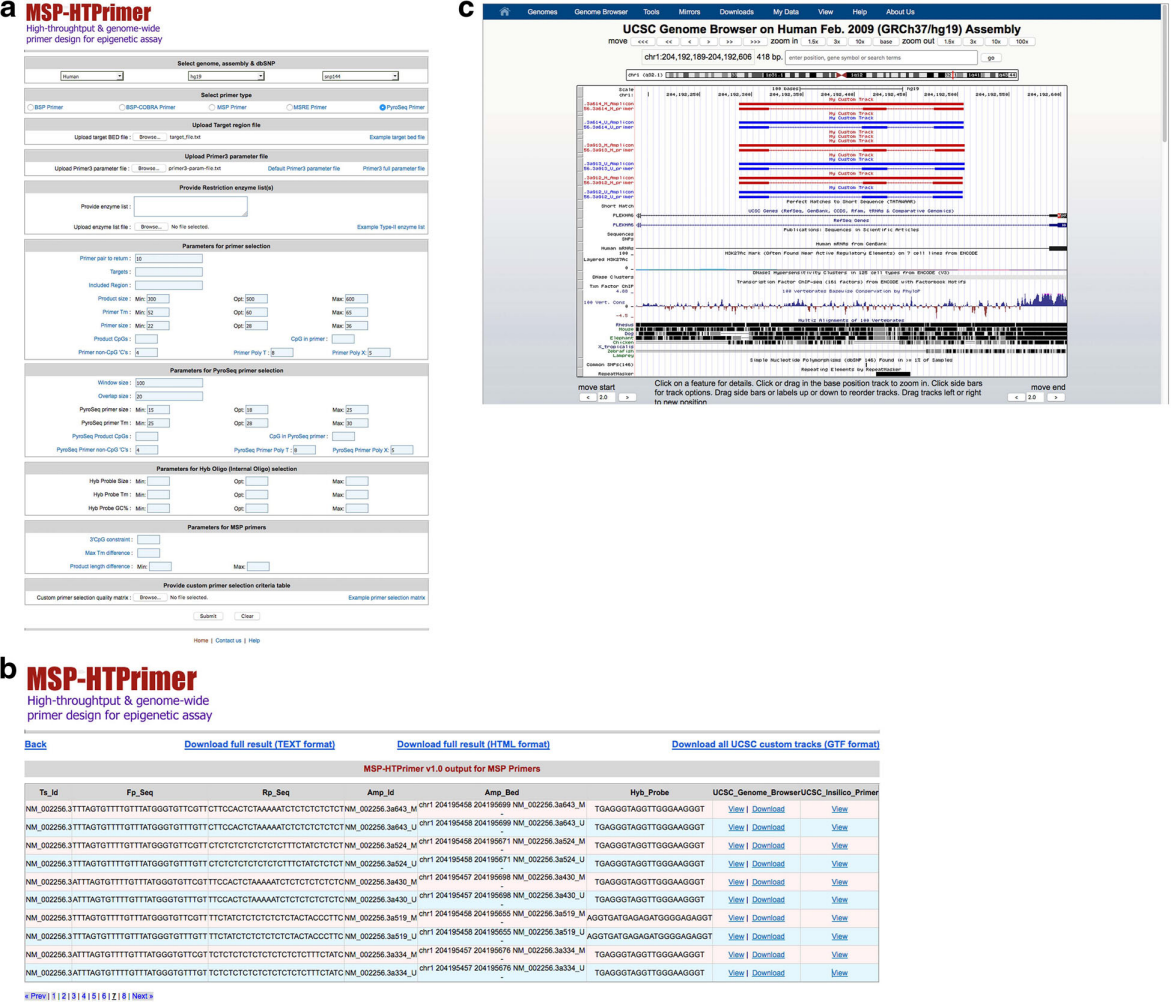
The MSP-HTPrimer web interface and output. **a** Web interface of MSP-HTPrimer query page. The query interface for MSP-HTPrimer shows different parameters that can be used to design and select optimized PCR primers for BSP [default option], MSP, COBRA, and MSRE assays; **b** an example of primer pair summary output table for MSP-PCR in MSP-HTPrimer web interface (for MSP primers two rows with alternate colors are displayed in *light red color* and *light blue color* backgrounds are for methylated and unmethylated primer pairs, respectively. The primer summary table contains target sequence ID, forward and reverse primer sequence, amplicon coordinates in BED format, hyperlink to visualize in UCSC genome browser, and in silico PCR database; and **c** an example output of the MSP-HTPrimer primer pair visualization in UCSC genome browser along with genomic (RefSeq, CpG islands, SNPs, and repeats) track within in BSP-HTPrimer web interface (For MSP primer, two tracks are displayed [*red color* for methylated primers and *blue color* for unmethylated primer]. Each result of the primer design pipeline is presented bundled, once as single *red line* [full amplicon] and as a line emphasizing forward and reverse primer below)

MSP-HTPrimer offers a very intuitive, user-friendly, and powerful query interface (Fig. 3a). After starting the virtual machine, query page can be opened within the virtual machine via http://localhost/msp-htprimer. MSP-HTPrimer allows users to design primers for any number of target sequences in a single run. User can select the appropriate genome name, genome assembly, and the dbSNP database. For primer design, user uploads a target bed file, Primer3 parameter file (optional), type-II enzyme list for MSRE-PCR and COBRA-PCR primer, and custom primer selection matrix file (optional). Moreover, the user can customize several primer design and selection parameters to obtain specific and optimized assays.

### MSP-HTPrimer input

MSP-HTPrimer requires four input files:

1. Target BED file
  This file contains the genomic coordinates for all target sequences (one line for each target sequence). It consists of four tab-delimited columns: (1) chromosome, (2) start coordinate, (3) end coordinate, and (4) a unique ID for each target region. The file needs to be prepared without a column header (Additional file 1).
2. Primer3 parameter file
  This text file contains the default Primer3 tool parameters and values for the Primer3 tool (Additional file 2). It is optional and if not provided, MSP-HTPrimer will use default Primer3 parameters.
3. Restriction enzyme file
  This input file is only required for MSRE-PCR and COBRA-PCR primer design. Each line contains a restriction enzyme name and multiple enzymes are allowed in a single run (Additional file 3).
4. Custom primer selection quality matrix
  MSP-HTPrimer supports selection of primer pairs based on user-defined selection criteria. A custom quality-filtering matrix can be provided as input file. As shown in Additional file 4, user can define a set of selection criteria and rank them using a scale of 1–10. MSP-HTPrimer assigns these ranks to the primer pairs for all target sequences. If this input is not provided, then primer pairs are returned based on the Primer3 ranking. MSP-HTPrimer supports mathematical operators, including “>,” “<,” “>=,” “<=,” and “-.” Any column header of the MSP-HTPrimer output file can be used as a parameter. The primer quality level represents the hierarchical rank associated with each of the output parameters in its respective row. These selection criteria and the ranks can be defined based on the output table columns.

### MSP-HTPrimer output

MSP-HTPrimer produces a summary output file in two formats: (1) a tab-delimited text file and (2) a HTML output file (Fig. 3b), which contain one line for each primer pair along with all annotations including target sequence ID, amplicon ID, hybridization probe and genome amplicon coordinates, number of cut sites, number of SNPs, number of CpG islands, repeat regions, upstream and downstream RefSeq genes (including their distance with respect to forward and reverse primer), and a direct link to UCSC genome browser. For all five-primer design methods (BSP-PCR, PyroSeq primer, MSP-PCR, COBRA-PCR, and MSRE-PCR), the MSP-HTPrimer tool produces a uniform HTML summary output table, which facilitates an easy output handling and post-processing.

### Visualization of primer pairs

MSP-HTPrimer offers the visual display of assays in the UCSC genome browser along with genomic annotations, CpG islands, common SNPs, RefSeq genes, restriction enzymes, and other genomic information available in genome browser tracks (Fig. 3c). In addition, MSP-HTPrimer also provides the primer pairs and hybridization probe for each target sequence as a UCSC custom track file in GTF format (Additional file 5), which can be used for other analysis or can be visualized in other genome browsers. The custom track GTF file (Additional file 5) is created for each target sequence and can be downloaded from the summary output table (see Fig. 3b).

### Availability, installation, and usage

MSP-HTPrimer is a powerful, portable, and web-based tool, freely accessible to all researchers. It is available along with its intuitive web interface as a fully configured virtual machine (VM) at http://sourceforge.net/p/msp-htprimer/wiki/Virtual_Machine/. The virtual machine is configured to run without any installation and can be executed using Oracle’s VirtualBox system (https://www.virtualbox.org/). In addition to virtual machine, source codes for Linux (https://sourceforge.net/projects/msp-htprimer/files/Linux) and MacOS (https://sourceforge.net/projects/msp-htprimer/files/MacOS) are available, which can be easily installed on any Unix computer. A detailed user manual including description of inputs, parameters, outputs, installation dependencies, MSP-HTPrimer usage, and a detailed step-by-step description of the MSP-HTPrimer pipeline is available from https://sourceforge.net/p/msp-htprimer/wiki. A test data set is available at https://sourceforge.net/projects/msp-htprimer/files/test_data.zip.

### Performance evaluation

**Fig. 4:**
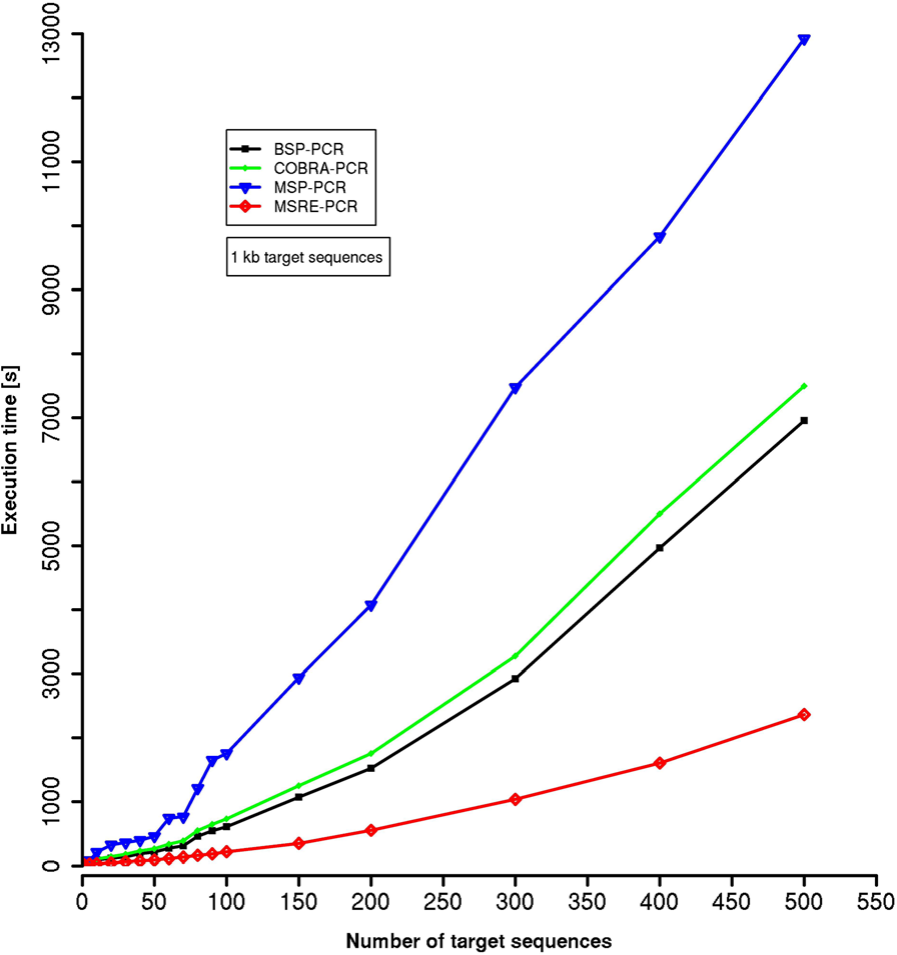
Evaluation of MSP-HTPrimer execution for BSP (black line), MSP (blue line), COBRA (*green line*) and MSRE (*red line*); considering number of 1-kb long target sequences. Different number of target sequences (1, 5, 10, 20, 30, 40, 50, 60, 70, 80, 90, 100, 150, 200, 300, 400, and 250 sequences of equal length of 1 kb)

MSP-HTPrimer is a high-throughput primer design pipeline and can design primers for one to several target regions simultaneously. To evaluate the performance of MSP-HTPrimer, from Human RefSeq genes (Hg38), we have randomly selected 500 target sequences of 1-kb length (±500 bp to the transcription start site) which fall within CpG island regions. The benchmarking was performed on a Linux server (Ubuntu 14.0.4 LTS with 8 CPU and 16 GB RAM). Execution times (seconds) were measured for all four methods BSP-PCR (black), MSP-PCR (blue), COBRA-PCR (green), and MSRE-PCR (red; Fig. 4). As shown in Fig. 4, MSP-HTPrimer is very fast and efficient to design-specific primer pairs for hundreds of target regions. As shown, the design for 100 MSP-PCR assays is conducted in nearly 1755-s (~29 min) computing time to run the entire steps according the pipeline. For the same dataset, MSP-PCR design takes more time than other methods (e.g. BSP - 608 s, COBRA - 731 s, and MSRE - 216 s for designing 100 assays), which is due to the design of a pair of primers for each the methylated and unmethylated target sequence, and checking the compatibility of primer pairs and their PCR products with respect to product length, number of CG, number of Cs, primer length, and CG position in primers (Fig. 4).

### MSP-HTPrimer experimental validation

We have experimentally validated the primers designed by our MSP-HTPrimer pipeline for methylation sites selected from a genome-wide discovery study using Illumina’s Infinium HumanMethylation450 BeadChip. Analysis of the BeadChip data of 48 twin pairs identified a set of 72 statistical highly significant CpG sites discordant for birth weight. That set was subjected to BSP primer design by using the presented MSP-HTPrimer pipeline. Predefined design and filtering parameters of the MSP-HTPrimer tool, such as the amount of number of CpG sites per assay, SNP filtering and avoiding position with repeats, combined with the standard parameters of Primer3 (e.g., sequence length, melting temperature, GC content, and primer length) yielded a total of 66 BSP assays. Sixty-three out of the 66 assays yielded specific amplicons under identical PCR conditions without further optimization (Additional file 6). These amplicons were generated for twins extremely discordant for birth weight (*n* = 48) and subjected to deep bisulfite amplicon sequencing using Thermo Fisher’s Ion Torrent PGM. Consequently, we have shown that MSP-HTPrimer designs primers and assays with very high success rates. Additional assay optimization steps (e.g., gradient PCR, adjustment of TAQ, MgCl2, adding of PCR enhancers) were only necessary for three assays.

### Intended user groups

Locus-specific methods, which are sensitive, specific, and cost-effective, are widely used to quantify the DNA methylation status. However, designing specific primer pairs is a first critical step for the successful DNA methylation analysis. For multiple genes, it is a cumbersome and challenging task to design several primer pairs to set up assays in laboratories with high success rate. In contrast to other bioinformatics primer design tools, MSP-HTPrimer’s flexibility and multiprocessing capability enables to design primers for thousands of targets in parallel. Moreover, it simplifies primer selection by applying efficient filtering based on a user-defined quality-filtering matrix. It allows users to develop optimized assays and thus significantly increases speed and success rate of assay design for all different PCR-dependent methylation analyses, suitable also for pyrosequencing and highly paralleled targeted deep bisulfite amplicon sequencing. The indented user group includes researchers which study DNA methylation by using the bisulfite modification- or MSRE-based approach.

### Comparison with existing tools

Today, several open source and commercial primer design software/tools are available including Methyl Primer Express, MethPrimer, BiSearch, MethMaker, MSPprimer, Bisprimer, PrimerSelect, Batchprimer3, PrimerPremier, and PRIMEGENS. The comparison of the MSP-HTPrimer pipeline to each of these tools is shown in Table 1. In total, 20 different points including availability, operating system, and installation requirements as well as necessary dependencies, multiprocessing capabilities, limitations of input file size, target sequence length, number of target sequences, visualization of results in UCSC genome browser, and genomic annotation of results were evaluated (Table 1). MSP-HTPrimer is available for free and can be run on any operating system. Furthermore, it is available as a fully configured virtual machine and thus has no installation requirements. MSP-HTPrimer has no limitations concerning the input file size, the amount of target sequences, or the target sequence length. Moreover, its multiprocessing capabilities dramatically reduce the execution time for high-throughput primer design. In addition, MSP-HTPrimer provides several improvements in comparison to existing tools such as the following: (1) primer design on both forward and reverse strand for bisulfite modification-based assays; (2) assigning genomic coordinates to all resulting primer pairs, oligos, and amplicons; (3) annotation of all resulting assays and amplicons with SNPs, RefSeq genes, repeat elements, and CpG islands; automatic FASTA sequence preparation based on the input target BED file (Additional File 1); (4) positioning of restriction enzyme cut sites; (5) visualization of results in the UCSC genome browser; and (6) produces output in commonly useful format (tab-delimited TXT and HTML table). Finally, MSP-HTPrimer is a one-stop pipeline, which will be very useful for bisulfite methylation-based (BSP, MSP, and COBRA) PCR, and methylation-sensitive restriction enzyme-based PCR (MSRE-PCR) primer design.

**Table 1:** Comparison of various features of MSP-HTPrimer and other tools for primer design

Features	MSP-HTPrimer v1.0	PrimerExpress v3.0.1	PrimerSelect	PrimerPremier	MethPrimer	PRIMEGENS-v2.0	BiSearch
Genome-wide primer design	Yes	No	No	Yes	No	No	No
Primer design on reverse strand^a^	Yes	No	No	No	No	No	No
Dependency	No	Yes	Yes	Yes	No	Yes	Yes
Installation required	No	Yes	Yes	Yes	No	Yes	No
Operating system	Web-based	Windows	–	Windows, MAC	Web-based	All Unix, Windows	Web
Genome coordinate information^a^	Yes	No	No	No	No	No	No
SNP annotation^a^	Yes	No	No	Yes	No	No	No
Repeat element annotation^a^	Yes	No	No	Yes	No	No	No
CpG island annotation^a^	Yes	No	No	No	Yes	No	No
RefSeq gene annotation^a^	Yes	No	No	Yes	No	No	No
Restriction enzyme type-II cut site identification^a^	Yes	No	No	No	Yes	No	No
Multiprocessing capability	Yes	Yes	No	Yes	No	Yes	No
Multiple target sequences	Yes	Yes	No	Yes	No	Yes	No
Target sequence number restriction^a^	No	Yes	Yes	Yes	Yes	Yes	Yes
Target sequence length restriction^a^	No	Yes	Yes	Yes	Yes	Yes	Yes
FASTA sequence selection by tool	Yes	No	No	Yes	No	No	No
Custom primer selection quality matrix^a^	Yes	No	No	No	No	No	No
Input file limitations^a^	No	Yes	Yes	Yes	Yes	Yes	Yes
UCSC genome browser visualization^a^	Yes	No	No	No	No	No	No
Availability	Free	Commercial	Commercial	Commercial	Free	Free	Free

^a^Features unique in MSP-HTPrimer

## Conclusions

We report MSP-HTPrimer, a web-based, robust, and one-stop high-throughput primer design pipeline for bisulfite deaminated and MSRE-based PCR locus-specific DNA methylation assays with multiprocessing capabilities. MSP-HTPrimer annotates all resulting primer pairs by adding genetic and epigenetic information including SNPs, RefSeq genes, repeats, and CpG islands, based on UCSC annotation tracks. It enables primer design for hundreds of target sequences on both strands (forward and reverse) in a single run based on customized design parameters. Bisulfite-based assays are considering both, the plus and the minus strand. MSP-HTPrimer has no limitation on the number and size of target sequences and provides full flexibility to customize the Primer3 parameters for specific requirement of each of the different assay concepts. Furthermore, it offers the opportunity to rank primer pairs based on task-specific preferences using a custom quality filter matrix in addition to general Primer3 ranking. In comparison to other tools, MSP-HTPrimer stands out for high-throughput and optimized epigenetic primer design capability and efficient primer selection for MSP, BSP, COBRA, and MSRE assays visualized in the UCSC genome browser.

## Methods

### Pipeline development

The MSP-HTPrimer pipeline was developed using Python 2.7.12 (http://www.python.org) and Biopython (http://biopython.org) with a special focus on multiprocessing capability to design five types of primers (1) BSP, (2) PyroSeq, (3) MSP, (4) COBRA, and (5) MSRE in a high-throughput manner. The reference genome FASTA sequence and annotations are used from UCSC genome browser, which are automatically retrieved and prepared by MSP-HTPrimer. The MSP-HTPrimer workflow is depicted in Fig. 1 consisting of eight sequential steps and starts with an arbitrary list of target regions and outputs a list of annotated PCR assays.

### Web interface development

The MSP-HTPrimer web interface was developed using HTML, Perl, and CGI and runs on an Apache web server. The graphical display of designed primer pairs and products for all target sequences are visualized in the UCSC genome browser. Hence, MSP-HTPrimer uniquely depicts the designed primer pairs, hybridization probes, and amplicons in the UCSC genome browser along with genomic annotation, restriction enzymes, repeats, conservation, RefSeq genes, and other information available in UCSC genome browser.

### Experimental validation of MSP-HTPrimer

An existing epigenome-wide study using the DNA methylation arrays from Illumina (Infinium HumanMethylation450 Bead Chips, Illumina, CA, USA) was used for experimental validation of the primers designed by MSP-HTPrimer pipeline in the lab. The samples for the study were deaminated using the EZ DNA methylation kit from Zymo Research, according to the manufacturer’s protocol and Illumina’s additional requirements. The Infinium assay provides distinct information about the methylation level of 485,577 cytosine sites per sample at single-cytosine resolution, whereas the interrogated cytosines are distributed over the whole genome. Based on human DNA methylation data (data not shown), a panel of 72 target regions was identified and selected for BSP primer design using MSP-HTPrimer software (Additional File 4). The BSP design parameters were set as follows: no SNP within the primer sequence and no common repeats within the assay. Furthermore, each original target position has to be located inside or close (±50 bp) to the PCR sequence. Assays were tested using an endpoint PCR (95 °C for 15 min, followed by 40 cycles of 95 °C for 20 s, 59 °C for 20 s and 72 °C for 40 s, and an final elongation at 72 °C for 7 min) with subsequent gel electrophoresis. PCR was set up in 10-μl reactions consisting of 1 μl 10× Taq Buffer (Qiagen, Hilden, Germany), 0.06 μl HotStar Taq (Qiagen, Hilden, Germany), 0.8-μl dNTP mix (each dNTP at a concentration of 2 mM), 6.14 μl H_2_O and 2-μl DNA solution (10 ng DNA/μl). Specific lanes on the gel and absence of primer dimers and/or artifacts indicate properly designed BSP assays suitable for TDBS. The experiments of the presented work were conducted under the FP7 project *EurHealthAgeing* and ethical issues have been approved by the NRES Committee London—Westminster under the study title *TwinsUK* REC Ref EC04/05.

## Additional files

Additional file 1: Target bed file (mandatory input file) to run primer design with MSP-HTPrimer. (TXT 74 bytes) Additional file 2: Primer3 parameter input file. This is the parameter for Primer3 tools to design primer pairs. Users can change these parameters to optimize and better positioning of the primer pairs for target region. These parameters are directly supplied to Primer3 tool. (TXT 478 bytes) Additional file 3: List of restriction enzymes (mandatory input for COBRA-PCR and MSRE-PCR) in a text file one enzyme per line. (TXT 41 bytes) Additional file 4: Hierarchical selection quality matrix with ten quality levels ranking the designed primer independent of the Primer3 level. (XLSX 25 kb) Additional file 5: An MSP primer UCSC custom track in GTF format produced by MSP-HTPrimer for each target sequence in a separate file. (GTF 2 kb) Additional file 6: Detail description of 66 experimentally validated BSP assays designed by MSP-HTPrimer. (XLSX 23 kb)
